# Supplementary material for: Fabrication of SnO2 Composite Nanofiber-Based Gas Sensor Using the Electrospinning Method for Tetrahydrocannabinol (THC) Detection
Source: Micromachines (Basel). 2020 Feb 12;11(2):190. doi: 10.3390/mi11020190 (PMC7074642; doi:10.3390/mi11020190)
Supplement: Supplementary file 1 [file micromachines-11-00190-s001.pdf]

Supplementary Materials

Fabrication of SnO_2 Composite Nanofiber-Based Gas Sensor using the Electrospinning Method for Tetrahydrocannabinol (THC) Detection

Pouria Mehrabi, Justin Hui, Sajjad Janfaza, Allen O'Brien, Nishat Tasnim, Homayoun Najjaran and Mina Hoorfar *

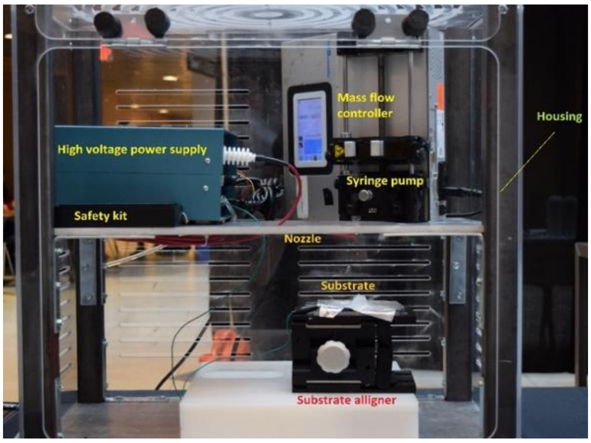

Figure S1. The image of electrospinning setup, including the power supply, housing, syringe pump, mass flow controller, and substrate aligner.

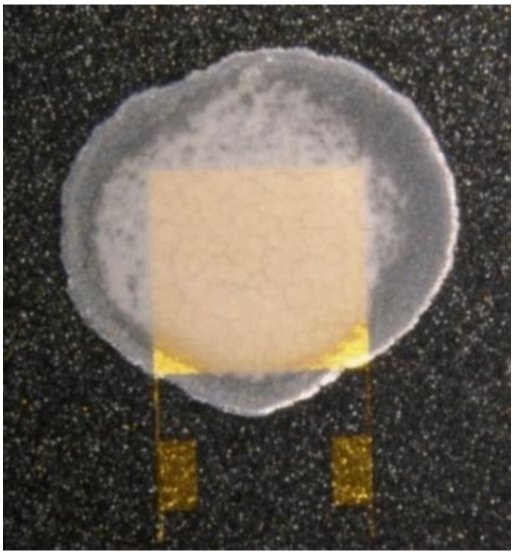

Figure S2. The THC sensor fabricated based on the SnO_2 composite nanofibers.

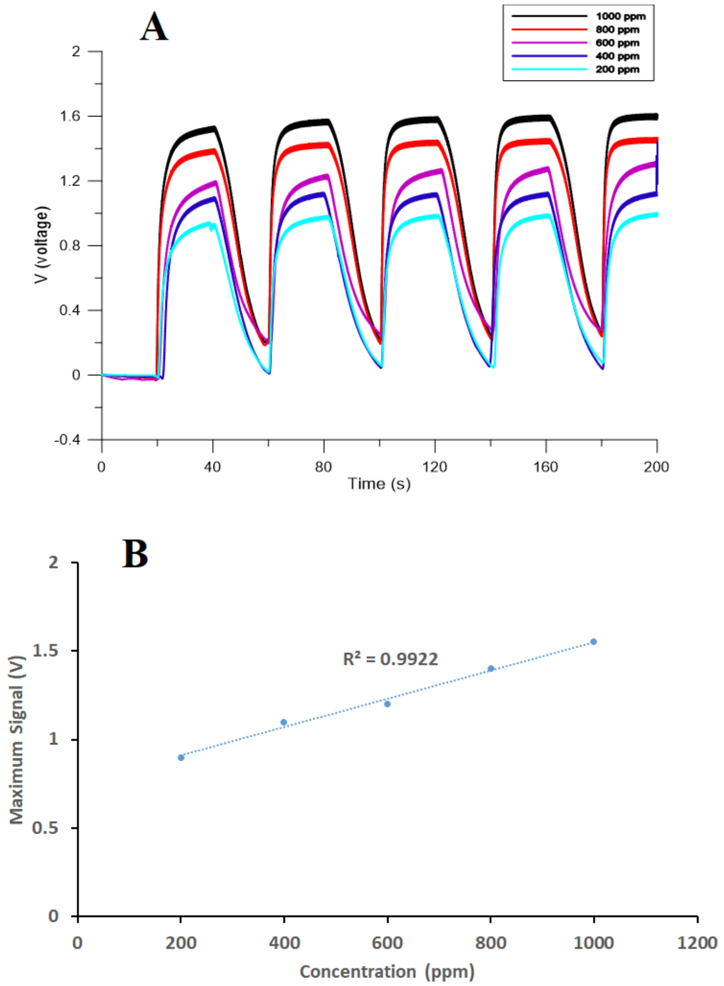

Figure S3. (A) The cyclic response of the sensor to 200,400,600,800, 1000 ppm of the mixture of methanol and THC, and (B) the calibration curve obtained from the response of the sensor to different concentration of THC.

Table S1. The percentage composition of each element in the deposited layer (10 nm gold deposition).

Spectrum Label	Spectrum Percentage
C	24.11
O	4.32
Al	34.02
Au	11.01
Cl	12.31
Sn	14.24
Total	100
